# Constructing self-rated oral health among disadvantaged older African Americans

**DOI:** 10.3389/froh.2026.1726866

**Published:** 2026-08-14

**Authors:** Mark R. Luborsky, Candace Ziglor, Divesh Byrappagari, Jocie Osika, Kimberly Shay, Judith Jones

**Affiliations:** 1 Wayne State University Institute of Gerontology, Detroit, MI, United States; 2 Department of Anthropology, Wayne State University, Detroit, MI, United States; 3 University of Detroit Mercy School of Dentistry, Detroit, MI, United States

**Keywords:** disadvantaged older persons, life course, life course/childhood circumstances, qualitative, self-rated oral health

## Abstract

Poor oral health care is prevalent among minority, disadvantaged, and older populations despite being readily treatable by effective, low-technology, low-cost interventions for prevention and early care. Oral health is a recognized priority ( 1, 2) in the goals set by the “Oral Health in Healthy People 2030″ (3) and the World Health Organization's call to for universal oral health coverage (4). However, many challenges exist, including inadequate awareness, knowledge, and access, particularly among disadvantaged groups. A key to improving use of services is knowledge of the individual's own appraisal of their oral health. Today, our understanding of these appraisals remains inadequate. This article offers insights into how older Detroit inner-city residents participating in a community-based oral health equity project construct their oral health self-rating, oral health perceptions, and beliefs. Such information is critical to guide providers and policymakers working to improve oral health.

## 1 Introduction: poor oral health—overall health and well-being suffer

Good oral health is essential for health and positive aging experiences, while poor oral health can significantly diminish wellness. All-cause mortality in prospective cohort studies was independently associated with self-reported fair or poor oral health (5, 6). In later life, poor oral health increases the risk for multiple serious health harms, including impaired cognition, heart disease, cancer, and increased disability and quality of life (7, 8). Tooth loss increases the risk of cognitive decline (OR=1.23) and dementia (OR=1.13). Gum disease, periodontitis, afflicts 60% of people over age 65, and over 1 in 5 cases go untreated (9). It is a common cause of tooth loss; 15%–20% of older adults have lost all their teeth (10). Poor oral health negatively impacts quality of life, the ability to socialize without pain or embarrassment, and social participation (1, 11). Substantial inequities exist with higher rates among low socioeconomic and minority groups. Disparities in dental attendance are particularly in high- and middle-income countries, where dental care is often concentrated in affluent areas, leaving vulnerable groups underserved (2). In the US, Blacks, Hispanics, and Asians have significantly lower oral health knowledge compared to Caucasians (12–14), and in later life.

Affordability affects access leading to financial burden (15). While many older adults report having some form of dental insurance, coverage is often limited and may not adequately cover restorative, prosthetic, or maintenance care. For disadvantaged older adults, premiums, copayments, uncovered services, and benefit restrictions can leave needed oral health care effectively unaffordable despite nominal insurance coverage. Inner-city Detroit low-income and disadvantaged residents face a federally documented shortage of available dental care: Michigan's 2025 State Oral Health Plan reports that HRSA-designated dental provider shortage areas include “much of the city of Detroit”; nearly all such designations apply specifically to low-income residents (16). This shortage is reflected in patient experience: dental care was the greatest unmet healthcare need in the city, with 14.2% of Detroiters reporting that they wanted but could not obtain dental care (17).

Providing oral healthcare to older adults presents challenges. Fewer facilitators than barriers were identified in a systematic review of challenges to providing oral health care to older adults (18). The main facilitators were the dental professional's regular presence and care regulations. According to dentists, care managers, and informal caregivers, the most frequent barrier was a lack of suitable facilities or transportation for patients. Barriers included a lack of knowledge about what constitutes oral health care, limited capability, refusal of care (55% each), and caregivers’ lack of time or skills (41%). These factors are especially consequential for disadvantaged urban older adults, for whom oral health perceptions and experiences are often shaped by cumulative inequality.

Relatively scarce literature is available on older adults’ understandings and experiences of oral health, especially among those at risk. Systematic insights into the nature of older minority adults’ self-ratings of oral health are needed. Such data can improve the effectiveness of interventions and policy. Noting the scant qualitative literature, Macdonald (19), among others (20, 21), called for qualitative insights into the nature of an individual's own oral health perceptions, understandings, concerns, experiences, and the influence of social settings, structures, and political contexts that shape self-rating of oral health. We know from the few qualitative studies of patient perspectives on periodontal disease that older patients express shock and surprise at the condition and the potential for tooth loss upon diagnosis (22). Patients believed the condition was a natural part of aging and poor oral health practices. Interviews with community-dwelling older adults (23) described the need for more nuanced tailoring of oral health information and knowledge, better access to care, greater affordability, reduced distress during dental visits, improved relationships with dentists, and slower change in attitudes and practices. Themes emerging in other qualitative studies include dental education specific to issues and communication with older persons, needs improvement, financial concerns, life course experiences from childhood fear and pain likely before modern dentistry (shared history of mistreatment in healthcare among minorities (24). Experiences of stigma and regrets were expressed by people with periodontal disease, in addition to impairments in daily physical, psychological, and social life (25). The authors suggest that greater attention to these dimensions is necessary to enhance the management of patients with periodontitis.

To conclude, oral health problems lead to widespread, preventable suffering despite the availability of interventions that are highly effective, low-cost, and technologically simple. The objective of this study is to address the knowledge gap regarding the contents shaping patients’ rating of their oral health and experiences, which may help providers and policymakers align with patient perceptions needed to improve care-seeking.

## 2 Materials and methods

This qualitative cross-sectional study was conducted in collaboration between Wayne State University's Institute of Gerontology and the University of Detroit Mercy School of Dentistry's Senior Oral Health Equity Project (SOHEP), a place-based program aimed at examining and improving oral health among disadvantaged older populations in Detroit who endure a disproportionate yet preventable burden of pain, disability, and illness due to poor oral health. The program is delivered at three locations in Detroit where seniors congregate or live, providing screening and preventive procedures, denture adjustments, fluoride treatments, and education. It also helps train future dentists and hygienists. Interviews were conducted with attendees to gain insight into their perspectives, experiences, and beliefs.

### 2.1 Methods

Recruitment was conducted sequentially on-site after completion of the SOHEP services. Recruitment for a purposive sample (26) was designed to assess the meanings and experiences of oral health. IRB approval was obtained (University of Detroit Mercy IRB #2122-09).

Following US Common Rule 45 CFR 46.117(c), a waiver protecting participant confidentiality, and consistent with our IRB-approved procedures, participants received an information sheet and provided verbal consent; signed consent forms were not collected, which would create identifiable links to participants. Consistent with this approach and given the small sample size and the specificity of the clinical context, we do not report individual-level descriptors alongside quotations, as such descriptors could allow disclosure. Aggregate sample characteristics are reported in Table 1.

**Table 1:** Characteristics of participants.

Characteristic	Category	%
Age	55–64 years	12.1%
	65–74 years	55.7%
	75 years or more	32.2%
Sex	Male	21.0%
	Female	79.1%
Ethnicity	African American	88.9%
	White Caucasian	11.1%
Dental Insurance	No	29%
	Yes	71%
Last Dental Visit	< 2 years	57%
	2–5 years	29%
	> 5 years	14%
Dentition	Mean number of teeth	14
	Functional dentition < 20 teeth	40.0%
	Functional dentition ≥ 21 teeth	60.0%
Treatment Urgency	No obvious problems	7.0%
	Early care needed	37.0%
	Urgent care needed	57.0%

### 2.2 Sample

Inclusion criteria were receipt of SOHEP services and willingness to participate in an interview; exclusion criteria were failure to meet inclusion criteria.

Sample size evaluation indicated that 16–24 interviews would be adequate; the final sample of 62 met this target. This size follows guidance for qualitative interview studies, where for a shared group condition, thematic saturation occurs at roughly 10–12 interviews and meaning saturation at 16–24 interviews (27, 28). The final sample included 62 people; overall, 73 people were approached, and 11 refused (e.g., could not stay, not interested).

### 2.3 Data collection and anthropological analyses

Interviewers were trained to conduct semi-structured interviews using an IRB-approved guide that facilitated discussions and storytelling.

The project was inductive, using open-ended discussion with some semi-structured prompts. Interviewer preparation was additionally guided by a review of “sensitizing concepts” (29–32), informed by an anthropological life-course perspective (33, 34). This discovery-oriented inductive approach was not based on a fixed *a priori* codebook or deductive strategy (29, 35). Discussion topics included oral health knowledge and perceptions, current and earlier life experiences with dentists, and sources of knowledge. Interviews were transcribed verbatim.

To determine how self-ratings of oral health are constructed, we used the Cognitive Interview debriefing method (36, 37) to discover and describe how participants interpret and process the wording of the question and answer options, and to document the rationales and knowledge they use to select a response. It is an open-ended discussion strategy rather than a conceptual framework.

After verbatim transcription, ML, JO, and KS conducted ethnographic content analysis. They first read each interview repeatedly to become familiar with the participant's account, then to identify topics, themes, and patterns (38, 39), using constant comparative methods (40). Then, codes were developed, initially using participants’ words and later using researcher-assigned codes.

Analytic themes were developed through rereading to identify and compare differences and similarities. We grouped codes with similar features into higher-level categories and then rereviewed, defined, and named them (38, 41, 42). For example, the participants discussed their earlier dental experiences and how those shaped their current oral health self-rating, which we termed the “life course context.” Building on the initial set of core categories and subcategories identified in the analysis, we conducted a further review. When differences in interpretation arose, these were addressed iteratively through discussion and the comparison of alternative readings of the original transcripts and coded excerpts, until agreement was reached on the most analytically defensible interpretation. These discussions focused particularly on category boundaries, thematic organization, and the data supporting the manuscript's interpretations.

We condensed some and discarded others that were not consistently relevant to the study's aim. Finally, four core categories were conceptualized, each with properties that characterize the main components of oral health experiences and beliefs, as explained in the results section. Overall, developing a higher-level conceptualization of categories was an iterative process aimed at refining the fit with emerging concepts in the data. Qualitative analysis software was not used.

Saturation was evaluated based on the richness of meaning and the depth of knowledge, rather than simply recognizing the theme's existence (27, 43–45). Strategies used to ensure trustworthiness included triangulation, reflexivity, maintaining an audit trail, and peer debriefing” (46, 47). First, the analysis relied on constant comparison across interviews and across categories to assess the consistency and variation of emerging interpretations. Second, peer debriefing occurred through analytic review by coauthors who collaborated in team meetings following independent examinations of the developing coding structure and thematic organization and challenged interpretive assumptions. Third, the team used reflexive practice by considering how disciplinary training, prior oral health research experience, and interviewer roles could shape both data generation and interpretation. Fourth, coding files, category revisions, and memos served as an audit trail documenting major analytic decisions. Together, these procedures (48) enhanced the credibility, dependability, and confirmability of the findings.

## 3 Results

The sample was primarily African American (88.9%), female (79.1%), and aged 65–85 years (Table 1). Dental exams revealed that over 40% had fewer than 20 teeth, and 90% of dentate individuals had untreated decay. Over one-third (37.3%) reported avoiding eating certain foods due to poor oral health, and a significant number (43.3%) had not visited a dentist within 2 years, despite most participants indicating they had insurance (49). The mean number of teeth was 14, which is below the US national survey reports of 16.7 for this age group, Black, and disadvantaged populations (10, 50).

### 3.1 Self-rated oral health—adults explain and construct their response

We merged replies into two categories (excellent/good; fair/poor) based on extensively replicated findings (51) on the self-rated health item, which show that a 2.5- to 3-fold greater likelihood of all-cause mortality is independently associated with fair or poor ratings compared with good or excellent ratings. In our sample, over half (61%) self-reported Fair or Poor oral health, and 39% reported good to excellent oral health. These percentages somewhat underestimate the objective status of the sample, given that clinician exams found that roughly 90% had undiagnosed tooth decay. The topics elicited during the cognitive appraisal process differed between the two categories in terms of focal content. Responses for each are presented in Table 2. Excellent, very good, and good self-reported oral health explanations focused on regular professional and daily oral care, with no pain or soreness, no gum swelling (periodontitis), no bad breath, and treated cavities (fillings). Social comparisons included appraising their oral health as good relative to others their age, with no mention of insurance or costs. In contrast, fair and poor oral health were attributed to costs, missing teeth, pain, difficulty eating, denture problems, and unpleasant breath—inadequacies or discomfort with professional or personal oral health care featured in these self-reports. A history of negative experiences with dentists also emerged as a rationale.

**Table 2:** Self-rated oral health explanation topics.

Topic	Excellent/Good	Fair/Poor	Exemplars
Natural teeth	X		"Well, I had my teeth pulled. So. I guess it's excellent." "Haven't had any cavities. And I used to have gum disease in that. And it's improved a lot, according to the dentist, and I had fractured teeth, and I taken care of those."
Dentures, bridges	X		"My gums, my tongue those parts excellent condition. I have some dentures; makes it even better."
Adequate function	X		"I have enough teeth to chew."
Pain absent	X		"I don't have pain, my mouth is not sore." "Because I don't have any problems. I don't have any swellings on my gums. I don't have any canker sores or anything like that."
Pain present		X	"I cannot tell a lie. So yeah, I mean, I have a lot of what do you call these things? Bridges? My top teeth? Too many. I should have taken better care. I've been getting kind of pain."
Social interactions	X	X	"My boyfriend when I get in the car he makes these weird gestures with his nose, can you smell my breath, and around people they would do like this, they really hurt me." "Partials are broken can only chew on one side. I'm only out talking to people because I can wear this mask (COVID). Without a mask I'd be a bit embarrassing."
Need dental care	X	X	"I have plaque. I don't get treatment. Often. I need cleaning. I have not seen a dentist and over six months to a year. And I don't brush my teeth more than once a day. And I eat sticky foods. So I think my overall oral health is poor." "I had to either come up with the money or just do without. So that's why I said, fair. I wasn't able to afford to just go to a dentist. And it went downhill with my dental."
Negative dental experience		X	"Unpleasant experiences with dentists. I am apprehensive about going unless I really need to. And I haven't been in three years. So I know I need to go. Like today my dentures were cleaned. Already I can feel the difference. I know I should be going more frequently. But I've had these two bad experiences with dentists. So if I'm not really hurtin’, I tend not to go."
Not self-care adequately		X	"I cannot tell a lie. I have a bridges in top teeth. I should have taken better care. I'm beginning to have problems with my top." "I don't have insurance; I've been looking for a dentist where I can afford to get my partials. . I'm thinking my dental health my overall health is poor, because I haven't had it taken care of like it should have."

### 3.2 Understandings of oral health

Understanding individuals’ attributions of causes, whether natural to aging or due to behaviors such as neglect, and the elements that feature in their self-rating of oral health, offers insights for addressing oral health issues in a more nuanced, individual-specific fashion, and how these considerations incorporate professional knowledge, offers clinically valuable insights. Interviews explored perceived causes and the course of oral health from a lifespan perspective and identified five categories of beliefs (Table 3). The naturalness of eroding OH was asserted by over three-quarters of the sample.

**Table 3:** Aging and oral health changes — A natural part of Old Age? (Yes 83%; No 16%).

Category	Percent	Example
No	12%	"No, if someone took care of the teeth, like my husband, 71, only missing one tooth. When you get older if you take care of your teeth, and get the right nutrients it shouldn't be that way."
No + Behavior	4%	"No its a natural consequence of habit, lack of good dental care, and our diets fast foods with high sugar content, that kind of thing."
Yes: Nature + Behavior	6%	"It may be natural as we get older. And our medical, medications we take a lot of that stuff."
Yes: Nature + Resources	5%	"Of course, because things gonna happen, your mouth shifts, you get decay, or gum decay, your gums might shrink down, and you might have to have new dentures, or you might not have the money to get new dentures. All of that affects it."
Yes	72%	"Everything breaks down the mouth got to break down too. It's nothing, you can preserve the process for a while. You can't stop it." "Most definitely. Most are deaf or blind, that's just part of life process."

### 3.3 Oral health analytic themes

Using a systematic iterative team-analytic process (see the methods section above), we identified four higher-order analytic themes: lifespan oral health pathways, knowledge gaps, barriers to care, and the role of non-clinical others in promoting oral hygiene. Overall, the findings suggest that oral health is shaped by multiple intersecting forces, including societal, political, economic, and historical factors that extend beyond individual behaviors.

#### 3.3.1 Lifespan and contextual factors shaping oral health pathways

Participants described various ways they learned about oral health care throughout their lives. Many cited parents or other family members from childhood as primary sources of knowledge, while others mentioned school programs or military service. One participant recalled his first realization about oral health care services and personal care, “When I went into the military in the late 80s, we had free dental care, so I started.”.

Discrimination and inequality-related painful experiences as an African American emerged in discussions, in a reluctant, but forceful fashion. Recounting stories of experiences seeking dental care earlier in life that resonate today, as a continuing hesitancy to seek oral health care.

“When you had problems when we were small [growing up poor and Black, they just pull your teeth. So they didn't get cavities filled. No proper dental care until in the Navy.”

“Unpleasant experiences with dentists. I am apprehensive about going unless I really need to. And I haven't been in three years. So I know I need to go. Like today, my dentures were cleaned. Already I can feel a difference. I know I should be going more frequently. But I've had these two bad experiences with dentists. So if I'm not really hurtin’, I tend not to go.”

Life course later life transitions, such as the death of a spouse, directly impact this population.

“When they cut the dental programs for spouses, I had to come up with money or do without. I wasn't able to afford to go to a dentist. And it went downhill.”

#### 3.3.2 Knowledge gaps

Participants demonstrated varying levels of understanding regarding oral health. Oral health is attributed largely to personal oral care behaviors (58%), while 42% attribute it largely to the dentist's responsibility. Roughly 62% viewed oral health as including the entire mouth, while others focused solely on teeth or breath (28%).

Approximately 62% of the sample exhibited a relatively comprehensive understanding of oral health. Participants described oral health, for example, as:

(The) “structure of teeth and care of your gums, and also daily brushing and your teeth, and also using what is it the you know, the little string thing? What is that called? Floss, the floss, floss, you know, flossing, all those things are part of oral health.”

“That is when you are taking good care of the entire mouth, teeth gums, the whole mouth”

In contrast, other participants’ understandings of oral health did not incorporate the entire range of clinically defined elements. Their accounts encompassed fewer components than those specified in the full clinical definition of oral health. For example:

“When my breath smells good without anything coming from rotten teeth”

“Not having bad breath”

These represent a highly sensory and experientially based perspective on oral health, similar to others who are concerned that periodontal disease remains a “silent condition,” one that is not readily amenable to individual recognition or diagnosis.

Yet others reported that poor oral health is a natural part of aging itself or of later-life experiences (see Table 3). “Everything breaks down the mouth got to break down too,” a view shared by more than three-quarters of these older inner-city Detroit African Americans.

## 4 Discussion

Our qualitative study describes the elements used by primarily African American older residents of inner-city Detroit to construct their self-ratings of oral health and articulate their oral health perceptions and beliefs. By using cognitive interview debriefing (36, 37) and life-course-oriented ethnographic analysis (33, 34, 38, 39), we unpack the heterogeneous rationales, experiences, and beliefs that underlie how individuals interpret the seemingly simple response categories of the standardized self-rated oral health (SROH) item. The study results highlight that self-ratings are shaped by an intersecting constellation of current clinical status, knowledge gaps, life-course events, financial and structural barriers, and experiences of discrimination and mistrust. In doing so, this study responds to calls for qualitative knowledge of the nature of oral health perceptions and self-ratings (19–21) and addresses the knowledge gap regarding how older adults, in their own words, interpret and explain their oral health self-ratings, offering empirical detail that can guide both clinical care and public health efforts.

Several of our findings align with prior work while also extending it in ways that are particularly salient to disadvantaged and older adults. Consistent with other studies, many participants framed oral health decline as a “natural” part of aging (52), echoing broader perceptions of disability and illness in later life as age-normative despite their treatability (53, 54), and cited financial constraints as a major barrier to accessing needed dental care (55, 56). At the same time, our data show how these beliefs are embedded in lifelong trajectories of unequal access and disadvantage, supporting cumulative inequality perspectives that emphasize how earlier exposures “get under the skin” and shape later-life health perceptions and behaviors (57, 58). The contrast between those who reported good or excellent oral health—often describing regular preventive care and the ability to function without pain—and those rating their health as fair or poor—who highlighted pain, missing teeth, eating difficulties, problematic dentures, and costs—shows how SROH captures both material conditions and the meanings assigned to them.

A main contribution of this study is its life-course and structural emphasis. Participants described formative childhood and young adult experiences—such as growing up in poverty, receiving extractions instead of restorative care, or encountering discriminatory and painful treatment—that fostered long-term mistrust of dentistry and a tendency to delay care until problems became acute (22, 24, 25). Later-life transitions, including the loss of spousal dental benefits or other structured coverage, were associated with abrupt declines in dental care use and worsening oral health. These narratives illustrate how structural factors and a mutual history of mistreatment among African Americans continue to shape both clinical status and care-seeking in older age and parallel patterns noted in other underserved and minority populations where dental services are concentrated in more affluent areas (2, 12–14). Taken together, the findings highlight that present-day SROH responses cannot be fully understood without considering the sociohistorical and institutional contexts in which oral health trajectories unfold.

Our qualitative data support conceptualizing SROH as a person-centered, lifespan, and socially situated appraisal rather than a report based solely on current biomedical symptoms. When forming their ratings, participants considered not only the presence of disease as well as pain, chewing and eating capacity, functional ability, appearance, breath, past experiences with dental professionals, affordability of care, and social comparisons with peers. For some, oral health was understood comprehensively as involving teeth, gums, and daily hygiene practices. In contrast, others’ focus was limited to sensory experiences such as breath, or normalized tooth loss and periodontal deterioration as inevitable features of aging (21, 23, 52). This pattern helps explain the gap we observed between subjective appraisal and objective clinical need—where 61% reported fair or poor oral health but 90% had untreated decay—because conditions that are painless, invisible, or perceived as age-appropriate may contribute little to negative self-ratings even when disease burden is substantial. Similar to self-rated general health, which is a well-established independent predictor of morbidity and mortality (51, 59), self-rated oral health has been shown to predict disability and mortality (60, 61), but our findings show that these single items condense biography, social comparison, structural constraints, and embodied experience into a short answer.

The findings suggest several implications for clinical practice and oral health policy, particularly in disadvantaged urban settings. Clinicians and community-based programs need to recognize that many older adults interpret oral health decline as a “natural” fact of aging and may underappreciate treatable problems; brief conversations that explicitly challenge fatalistic beliefs, normalize preventive visits, and connect symptoms to modifiable conditions may help reframe expectations (54, 55). Addressing these interpretive processes can help clinicians and program designers “meet patients where they are,” attending to both material barriers and entrenched beliefs that discourage care-seeking, particularly in populations that endure preventable oral health burdens. Education efforts should be customized to the ways participants actually talk about oral health—for example, building on sensory concerns about breath to introduce concepts such as gum disease and its silent progression—and should address the side effects of common medications on oral tissues in older adults (1). At the policy level, our participants’ experiences underline the importance of stable dental coverage for older adults, including the potential expansion of dental benefits within Medicare and sustained investment in community-based equity programs like the Senior Oral Health Equity Project (SOHEP) that provide low-barrier preventive care, education, and referrals in trusted local settings (16, 17).

These qualitative results also have implications for measurement science and the interpretation of self-rated oral health in population studies. Our data suggest that SROH responses reflect nuanced reasoning processes formed by life-course experiences, discrimination, and structural disadvantages, as well as current symptoms and clinical status. The frequent normalization of oral health decline as “what happens with age” points to a likely shift in response or recalibration, in which older adults adjust the standards they use to judge their oral health, an issue previously noted for self-rated health after serious health events in later life (62). This may contribute to underestimation of clinical needs, as illustrated by the discrepancy between reported fair/poor SROH and high levels of untreated decay. Very few studies have systematically investigated the reasoning processes underlying SROH responses (63–65), and our findings support more systematic use of cognitive interviewing and other qualitative methods in conjunction with psychometric testing to examine response processes, assess differential item functioning, and evaluate whether SROH categories carry comparable meanings among socially and clinically distinct groups (66, 67). Future large-scale studies could adopt mixed-methods validation designs in which brief cognitive-interview pretesting among disadvantaged subgroups informs item wording, interpretation, and analytic strategies before survey deployment.

Overall, this study supports a more valid understanding of how older, disadvantaged adults make sense of their oral health, strengthening the explanatory model of SROH while remaining grounded in participants’ accounts. Its theoretical contribution is modest but important: SROH should be viewed as a socially situated appraisal that integrates normalized aging expectations, embodied symptoms, life-course disruptions, structural barriers, and prior experiences of care or neglect, rather than as a transparent subjective report dictated solely by current clinical findings.

Beyond implications for individual practice, these data also correspond with and enrich equity oriented frameworks in oral healthcare. National and international initiatives from NIDCR, Healthy People 2030, and the World Health Organization have identified oral health inequities among older adults, low-income populations, and minority populations as critical targets for intervention. However, these efforts commonly rely on surveillance indicators that do not capture how affected groups themselves understand and appraise their oral health (1–3). By detailing how cumulative inequality, discriminatory care experiences, and the normalization of decline shape both clinical need and SROH responses, our qualitative findings supply context-sensitive information that can inform the design of equity-focused programs, including place-based initiatives such as SOHEP and similar community partnerships in other cities (16, 17, 19, 57, 58). Including such insights into planning and evaluation can help ensure that equity metrics and interventions are calibrated to the lived realities, expectations, and priorities of the older adults they are intended to serve. The following limitations emphasize important considerations for interpreting and generalizing these results.

### 4.1 Limitations

This study has several limitations. First, the data were drawn from a single city; further research is needed to compare these findings with those from other urban centers. Second, the sample consists primarily of inner-city, economically disadvantaged African American women and is not readily generalizable to other ethnic groups or to those with greater financial resources. However, focusing on this hard-to-recruit and hard-to-retain population reflects an explicit equity orientation: they endure a disproportionate yet preventable burden of oral health issues and remain underrepresented in research. Selection bias may also be present, as the participant pool was limited to those who chose to attend a free clinic and were recruited after receiving services; thus, gratitude bias or social desirability effects are possible. These are considered minor limitations, however, since discussions focused on participants’ personal life experiences and beliefs about oral health. Finally, because this is an older age cohort, the historically specific social, political, and structural conditions of Detroit that shaped oral health beliefs may not apply to younger cohorts.

### 4.2 Conclusion

This study reveals the multifaceted nature of self-ratings of oral health and perceptions among older Detroit residents. Addressing knowledge gaps, reducing barriers to care, and accounting for lifespan- and culture-specific influences are crucial to improving oral health outcomes in this population. Policy implications include the need for dental coverage in Medicare and in community-based programs that offer routine preventive dental care and referrals. Future research may benefit from mixed-methods approaches that combine clinical and qualitative data to explore further discrepancies between self-rated oral health and objective measures (19).

## 5 Consistency statement

We confirm that this manuscript was prepared as an Original Research article for Frontiers in Oral Health and reviewed for consistency across its aims, methods, findings, and interpretation. The Introduction frames the study around oral health perceptions, self-ratings, beliefs, and care experiences among older Detroit residents participating in a community-based oral health equity project. The Materials and Methods section describes a qualitative cross-sectional design using semi-structured interviews, cognitive interview debriefing, and ethnographic content analysis. The Results report findings that correspond to those methods, including participants’ explanations of self-rated oral health, understandings of oral health, and broader analytic themes concerning life-course experience, knowledge gaps, barriers to care, and the role of non-clinical others. The Discussion interprets these findings in relation to prior literature, measurement implications, intervention opportunities, and study limitations. Tables are used to support the sample description and thematic findings, and required statements concerning ethics, data availability, author contributions, funding, and conflicts of interest are provided in accordance with journal requirements.

## Data Availability

The datasets presented in this article are not readily available because data contain sensitive personal and community information that cannot be made publicly available, in accordance with participant consent and ethical approval. De-identified excerpts supporting findings are provided in the article. Limited, de-identified materials may be available from the corresponding author upon reasonable request, provided they align with the original consent, institutional ethics review, and a signed data use agreement. Data are stored on encrypted, access-controlled servers at the authors’ institution but not in a public repository. Requests to access the datasets should be directed to Mark Luborsky, mluborsky@wayne.edu.
